# Rabies, with a Report of Two Recent Outbreaks

**Published:** 1898-10

**Authors:** A. B. Kelly

**Affiliations:** Cohoes, N. Y.


### RABIES, WITH A REPORT OF TWO RECENT OUTBREAKS.

By A. B. Kelly, D.V.M.,
COHOES, N. Y.

Synonyms: Hydrophobia. *German*, Hundswuth; tollwuth; wuthkrankheit. *French*, Rage. *Italian*, Arrabioto; rabbia. *Spanish*, Rabioso; mal de rabia. *Roumanian*, Turborea. *Arabic*, Mkloub. *Greek*, Lytta; lyssa.

*History*. From a careful study of its history, it would appear that rabies was first described in the fifth century B.C. by Democritus[1] and a few other authors of that period. Since then the malady has been known as affecting different species, and in the second century B.C. it was stated[2] that Aesculapius, the ancient god of medicine, and his two sons discovered its transmissibility from animal to man. This they held as a family secret.

Pausanias, in his work, *Travels in Greece*, alludes to a story concerning Actaeon, in which he was torn to pieces by his fifty hounds when he surprised Diana at her bath, and says that the only explanation could have been that they were rabid. According to recent researches by many investigators, this may be doubted, as it has been demonstrated that rabies does not arise spontaneously, but only by inoculation from a previously affected animal.

Homer is also thought to have been acquainted with the malady, for in his *Iliad* he speaks of the "dog star," or "Orion's dog," as having a malignant influence on man.

Several of these early investigators spent much time on this most important subject, and each thought that he had at last located the seat of the disease, as follows: The stomach (Artemidorus[2]); the diaphragm (Magnus[2]); the meninges (Herophilus Cajus[2]).

In the first century B.C. Celsus undertook to clear up some of the allusions made by previous writers, and, as a result of his researches, concluded that the saliva alone contained the pathogenic material, and, therefore, recommended the cauterization, burning, cupping, or sucking of wounds received from any supposed rabid dog.

At this period every physician tried to devise some means of prevention or radical treatment for the people who had been bitten; consequently many queer and useless therapeutic measures were tried, and some were even adopted as specifics. Among these were hot baths. The victim would be placed in a hot bath and caused to sweat as long as the system would permit. Celsus[2] recommended hot or cold baths, to bind salt in the wounds, to throw a victim unexpectedly into the sea. Caustics, cupping, sucking, and bleeding were highly recommended, and are to-day.

Among the preventive measures advised were cauterization, suction, and bleeding (Celsus); tree-box (Hippocrates[2]); removal of the "sublingual lyssi" or worm in the tongue (Pliny[2]). Pliny also states that if this "lytta" is removed from a pup he will never lose his appetite or get mad; and if this worm is carried thrice about a fire, and given to some person, he will be immuned from rabies for one year and no more. He also alleges that the eating of a cock's brains will prevent the disease.

Some claimed that a pounded cock's-comb, or goose-grease mixed with honey, was highly efficacious as an application to the wounds. Others advised victims of any bite to drown a pup of the same sex as the one inflicting the bite, and to eat the liver raw.

In the enormous list were also found such remedies as to eat the salted flesh of a rabid dog; to apply the ashes of a dog's head to the wound; the application of a maggot from the cadaver of a rabid dog to the victim's body; to wear a dog's heart; to wear a dog's tongue under the great toe in the shoe; to drink the saliva from under a dog's tongue, etc.

Many other such fantastic alleged remedies and preventives were recommended, illustrating the absence of any rational idea as to the true nature of the malady. This affection was supposed to so poison the system that even the urine, if trod upon, would inoculate people, especially if they had any wounds on their feet, and for this they applied the feces from a horse sprinkled in vinegar and warmed in a fig.

One of the most useless of these prophylactic measures was proposed by Columella[2] in the first century A.D. It consisted of biting off the first articulation and withdrawing the tendons of a pup's tail on the fortieth day after its birth. It seems that the folly and cruelty of this operation should be thoroughly understood by this generation, but we are informed that it is still practised by the ignorant.

That the existence of the malady was well known by some of the early investigators is shown by the following symptoms, as prepared by Caelius Aurelianus[2], of Numidia, about the fifth century A.D. (?). He obtained his knowledge from Eudemus, Soranus, and others, and set forth his conclusions as follows: "Symptoms: The hydrophobia in man is preceded by extreme irritability, unusual motions of the body, disturbed sleep or absolute wakefulness, indigestion, stretchings, yawning, nausea, imaginary notions of bad weather, and no appetite for drink. To these symptoms succeed, when the hydrophobia begins, a desire to drink (a violent thirst), with terror at the sight, sound, or name of water. The patient is even afraid of fomentations with oil; his pulse is dense (hard), small, and irregular; there are sometimes a small degree of fever, convulsive motions of the stomach, spasms in the precordia, numbness of the joints, and torpor of the bowels; frequent inclination to micturate; trembling and catching of the limbs; voice hoarse, resembling the barking of a dog; spiral posture of the body, like that of a dog lying on the ground; anxiety when any person enters the room, as if apprehensive that he should bring water; redness of the face and eyes; body emaciated; the upper parts pale and perspiring, and the frequent involuntary ejaculation of semen."

Aetius[2], an ancient physician of Mesopotamia, is credited with having had an accurate knowledge of this disease, as he states that "dogs are naturally hot and dry; that the increase of temperature which the air acquires in the summer months causes them to go mad, and that this madness is called rabies." He also attributes the attacks to the variations in the atmosphere and violent thirst; but this is now known to be a delusion, as dogs do not become rabid from such agencies, but only by infection. We must, however, allow that dogs do become cross and savage from humidity and annoyance, but a bite from such a dog would be no worse than a bite from a normal dog—*i. e.*, the victim could not contract rabies from such a bite.

*Etiology.* Little progress was made in the knowledge of the cause of this or any other infectious disease until 1863, when Davaine[3] demonstrated that anthrax was due to a specific organism, and that by inoculating animals with this microörganism the disease in question can be reproduced.

We must admit that for a long time previous to this date it was quite generally acknowledged that certain diseases were transmissible by direct contact, but by what means was an unsolved question, and in the same way the cause of the origin of a disease in a new locality was also unknown. This led a great many to believe that all infectious diseases arose spontaneously, and were caused by certain degrees of food, unhygienic surroundings, or weather.

During recent years numerous cases of false hydrophobia, pseudo-hydrophobia, or lyssaphobia[7] have been recorded by some very prominent physicians. This might give rise to the theory of spontaneous generation, but in reality it is only an hysteria. At times people have been bitten by dogs, and as they neglected the case, and constantly thought of it, their nervous systems were brought into such a state of excitement that the victim actually developed symptoms of rabies, and in some few instances died from the effects. The genuineness of these cases has been disproved by the negative results obtained when animal inoculation was resorted to, and also by the length of the period of incubation in the individual attacked. Such cases have been cured by the so-called "mad stone," which is in reality any stone that one is able to obtain during such an attack and that the patient can be persuaded to trust. Cases are also reported where people have been reasoned out of these attacks and have made complete and rapid recoveries.[9] Some investigators state that about one-half the cases in human beings are of this type. Therefore, it is safe to say that one cannot positively assert that any case is one of rabies until he has proved it by animal inoculation. This also proves that we must have some specific agent as a cause of the malady.

The discoveries made by Pollender[3] and Davaine encouraged other investigators, and every one was then looking for the specific cause of each and every disease. Success was attained in a great many cases, but unfortunately the specific cause of rabies has not as yet been discovered. Several have succeeded in finding that the nervous system was the principal seat of the virus, and some have claimed to have isolated the germ, but this has not been certainly proved, nor is it generally accepted. The saliva and bronchial expectoration are also found to be very virulent, and it is by these that the disease is spread through the bites of rabid animals.

About 1880 M. Pasteur undertook to prove that rabies was a specific infectious disease, and accordingly made a great number of inoculations from the brains and spinal cords of rabid animals intravenously. This proved to be a very slow and tedious experiment, and was not entirely satisfactory, as some cases failed to develop the disease at all, while those that did develop it required such a long period of incubation, and invariably developed the paralytic form, that the learned investigator sought some more suitable way to produce the disease. He soon discovered that the virus in the encephalon was the most virulent, and accordingly he placed this virus in direct contact with its natural medium. To do this he found that it was necessary to trephine the cranium and inject the virus subdurally. By this method he found that he could, with almost a certainty, produce rabies within a very short time, incubation averaging about fifteen days; whereas, by the intravenous injection, the incubation lasted from several weeks to several months, and sometimes to years. At this time he found that the virus from the encephalon would invariably produce the furious form, while a virus from the myel would produce the paralytic or dumb form. He also discovered that it made no difference from what section of the myel the virus was taken, as its virulence was constant throughout its entire course, and that the disease could be produced from some of the larger nerves, such as the sciatic and pneumogastric. Infection through the milk or semen has not been proven. This series of experiments thoroughly agreed with universal experience, that rabies was caused only by the bite of a rabid animal or by some other means of inoculation.

Rabies, according to Prof. Law, is "an acute infectious disease in carnivora, transmissible by inoculation to all warm-blooded animals, marked by a long incubation, followed by fever, nervous disorders (intellectual, emotional, and motor), and terminating in paralysis; is characterized by optic illusions or delusions, and runs a short course, generally terminating fatally."

Hill[4] says, "This disease may be truly designated the scourge of the canine race; horrible in its nature, alike terribly fatal to man and beast."

*Geographical Distribution.* Practically speaking, rabies is found to be well distributed over the whole world, but it is generally conceded that it is more prevalent in the temperate zones, although a few places in these latitudes are said to be absolutely free from it. It is claimed that the disease has never been seen in Australia, but this is probably due to the fact that there is a very stringent quarantine of three months on all imported dogs at a cost of fifty dollars each. New Zealand, Van Diemen's Land, Azores, St. Helena, Hebrides, Scilly, La Plata, Tasmania, Hong Kong, and Argentine Republic are also said to be free from rabies.

One of the main factors in the prevalence of this malady in the temperate zone is that it is more thickly populated and contains a greater number of dogs than other zones; hence, a greater mingling.

Sex seems to have some influence, not that one sex is more susceptible than another, but that a female dog in season will attract much attention from the opposite sex, causing several males to follow her, and, in their quarrels to determine their superiority, one mad dog will generally succeed in infecting the whole group; hence, it has been stated[8] that the ratio is about seven males to one female. Food, habits, and breeds do not appear to have any causal relation to the disease.

*Seasons.* At present we have great dread of the hot summer months, and call them "dog-days," and in some places the law provides that dogs be muzzled during this period; but from carefully prepared statistics we find that there is a considerable increase of cases during the early spring months. Thus, Bouley[8] had 755 cases in December, January, and February; 857 in March, April and May; 788 in June, July, and August; and 696 in September, October, and November. This is due to the fact that spring and early summer is the rutting season for bitches.

Europe has long been more or less affected with rabies, but some sections seem to be more favorable to the propagation of the disease than others. France, upper Italy, and Holland appear to be the particular seats at present, while Spain, Portugal, and Germany appear to have very few cases, the control of the scourge in the latter country being due to the strict muzzle-law. There are a great many more cases reported annually in England than in Scotland. Sweden, Norway, Denmark, Russia, and Lapland were the seats of epizoötics of rabies during the years 1815 and 1824. It is widely spread in China, India, and Afghanistan. In Africa it is called mkloub by the Arabs. In Southern Africa the disease seems to be very rare, for only one or two cases of supposed rabies are reported. This is not the condition in Northern Africa, as it prevails in epizoötic forms in some of the countries. South America seems to be rather free from the malady, although it visited Chile in 1835, Peru in 1803, and Brazil in an epizoötic form. It has made great progress in the West Indies. Mexico is comparatively free from rabies, although it is known there. In 1768 Boston was the principal seat of an alarming epizoötic of rabies in dogs and swine, but we cannot trace accurately the source of the originally diseased animal. Since that time it has been more or less prevalent all over the continent, but more particularly in the New England and Middle States.[11] It is claimed to be unknown in Greenland, but supposed cases have been reported in the Arctic region.

*Susceptibility.* This is essentially a disease of the canine race, the dog, wolf, fox, and hyena being the most susceptible. It is, however, inoculable in cat, horse, cattle, sheep, goat, deer, guinea-pig, rabbit, donkey, mule, hinny, badger, marten, monkey, stag, antelope, prairie-dog, rat, mouse, chicken, pigeon, and man; in fact, all warm-blooded animals. The skunk, in certain districts, is said to be rabific; but this occurs only in circumscribed localities, and manifestly as the result of the introduction of rabies among the skunks of such localities.

According to species, it is determined that the canine are the most susceptible, the bovine coming next in order. Cattle rarely inoculate one another, the infection being spread by rabid carnivora.

The following[5] will give an idea as to the relative susceptibility of the various species: In Germany, in 1886, out of 578 cases of rabies reported, 438 were dogs, 3 cats, 5 horses, 92 cattle, 32 sheep, 7 pigs, and 1 goat. In Austria, in 1887, out of 858 cases, 769 were dogs. The same year the French authorities recorded 1643 cases in dogs. In Germany, in 1890, out of 708 cases reported, 590 were dogs, 98 cattle, 11 cats, and 9 pigs.

A bite from a rabid animal does not necessitate an inoculation, as the virulent saliva may have been wiped off on the clothing; or, perhaps, several animals bitten in succession, the last attacked being less liable to infection. A bite from a rabid wolf is generally more serious than one received from a rabid dog, as the former usually springs for the throat or face. It has been stated of the people bitten by rabid animals that of those injured on the face and shoulders, 80 per cent. would contract the disease; of those wounded on the thighs or hands, 50 per cent. would contract it; while of those bitten on the legs only 30 per cent. would develop rabies. A greater percentage of men and children are recorded[6] as having rabies, women being less subject to inoculation on account of their domestic habits and style of dress.

Zuill[5] writes, "Of all rabid animals the cat is the most dangerous; it jumps at the face and attempts to tear it with the teeth and claws. It no longer fears the dog, which it attacks boldly."

*Symptoms.* In the human family, as well as in the lower animals, nearly all investigators distinguish but two forms of this malady, namely, the furious and the dumb (paralytic and lethargic[8]).

The period of incubation varies from ten days to three months, and has been recorded[6] as long as five years, the variations being due to the individual susceptibility, the virulence of the virus, the amount of virus introduced, and the location of the seat of inoculation. If a virus be passed successively through several monkeys, sheep, or hens, it will become attenuated and greatly increase the period of incubation. On the other hand, if we pass it through several rabbits or guinea-pigs, it will be greatly increased in its virulence, hence considerably decreasing the period of incubation.

At the close of the period of incubation a person will be noticed to be rather nervous and irritable; the wound will be red, pruritic, painful, and have a burning sensation; he is very talkative, but avoids speaking of the disease; there will be a slight rise of temperature, 100–103° F., which may fall to subnormal prior to death; general pallor; anorexia; wakefulness, and frightful dreams.

At first the patient will be anxious to drink, but usually fails to accomplish this act, for as soon as the fluid comes in contact with the fauces it is expelled with force, and spasms of the muscles of deglutition and respiration set in.

This is often followed by a tetanic state, with marked episthotonus and temporary cessation of respiration. This may recur with an attempt to drink, or from a stimulation produced by a sharp sound, touch, bright light, breath of air, or the sound of water.

Later the sight, sound, or even the name of water causes terror to the patient, although cases are reported in which there is no hydrophobia—simply the hyperæsthesia and pain, accompanied by the burning sensation, hyperthermia, anorexia, etc.

Some cases are ushered in by severe spasms of the throat, with vomiting, a greenish-brown mixture being thrown up. There is a disposition to be alone, and surliness and hyperæsthesia alternate; satyriasis is marked; urine contains sugar, albumin, and occasionally blood. Repeated attacks rapidly lead to exhaustion and emaciation.

Pulse quick, irregular, small; respirations quick and shallow, a deep inspiration often inducing a convulsive attack; tenacious mucus accumulates in the mouth and fauces; articulation is thick. Later, the convulsive attacks increase in frequency, and death from asphyxia may occur during one of them.

Some cases progress very slowly, the eyes sink, brow sweats, lips become blue, and patient dies slowly from asphyxia. At times there is almost a complete arrest of convulsions prior to death, which takes place from exhaustion. The mind may remain clear to the end.

Occasionally man is stricken with a form of rabies which corresponds to the dumb form of the lower animals. It consists in a progress of the infection, causing paralysis (Landry's paralysis[6]), which attacks the limbs first, and gradually extends cephalad until it reaches the muscles of the neck and head.

We notice that the manifestations in the lower animals are essentially the same.

The dog, when first attacked, will often be noticed to be very friendly, especially to those well known by him; again, he will be morose and sullen; he will refuse food and water, and later will be noticed to be nervous and irritable, and when attacked with the furious form will have a tendency to leave home and take very long journeys. He will sneak along with his tail between his legs, head low, hair roughened, and snapping at imaginary objects. He has no fear of any animals or man, but until he is very much excited will obey his master at command, but will not remember anything for any length of time; the appetite will be depraved, choosing nails, hair, rags, paper, sticks, and cinders in preference to wholesome food; he has no fear of water, as he may be seen swimming streams; he will run about without any destination in view, and will bite anything in his path, but will seldom chase persons or animals unless they disturb him; he will seclude himself and lie down to take a sleep, which is broken by sudden howls and spells of barking; the lower jaw will droop; he will lick dirt, wood, stones, or any cold object; at first the sexual desires are greatly increased; the animal will lick and gnaw at the wounds in his body; the vocal cords become paralyzed, causing a marked change in the voice, generally described as a "howling bark," which is very harsh as well as characteristic, usually uttering three barks at one opening of the mouth; vomition is sometimes present. There is difficulty in defecation, which appears to cause intense pain. Respirations are seldom affected; pulse is increased, and temperature rises somewhat, but, as a rule, it falls subnormal before death. Eyes will roll wildly or strabismus may be present; conjunctiva may be congested, and forehead is drawn into wrinkles. The hyperæsthesia is a very marked feature, the animal starting at the slightest sound. A dog will shrink from a blow, but will bear it without a whine. Later on, paralysis sets in, being first noticed by the difficulty in swallowing, changed voice, drooping of inferior jaw, protrusion of tongue, and faulty coördination. Any excitement will cause the patient to go into convulsions, which are short at first, but as the disease progresses the intervals between the attacks become shorter, and the paroxysms more severe until the paralysis renders them impossible During this stage the victim will become rapidly emaciated, and in the last stages the eyes are staring, dull, and sunken. Course, from three to seven days; in rare cases, ten days.

In the paralytic form the hyperæsthesia and delirium, if present, are of such short duration that it is passed unnoticed, the paralysis being the first symptom recognized. There is a loss of appetite, but the patient does not eat foreign bodies. This form terminates fatally in two or three days; never more than five days.

Law[8] describes another form which is seen principally in pet dogs. He calls it the lethargic form, which is characterized by a profound apathy without absolute paralysis; neither fury nor paralysis is present, but the patient will simply lie about, and not pay any attention to threats, punishment, nor coaxing, and will refuse food or drink, remaining curled up until death relieves him, which is usually from the tenth to the fifteenth day.

The cat seeks seclusion, but is easily aroused, and will then use teeth and claws, inflicting serious wounds.

The wild carnivora will lose their natural fear, and will enter cities and villages and attack men or animals.

In cattle the same general symptoms are present: restlessness, anorexia, loss of power or rumination, anxiety, fear, bellowing, hooking at trees, men, or anything in front of them; horns may be fractured; constant pawing, eversion of upper lip, general wild appearance, conjunctiva hyperæmic, abundant salivation; constipation, feces in small lumps held together by a tenacious mucus; animal, when left alone, will stand quietly, with head low and eyes half closed, but at the slightest sound be in a wild state of excitement; if a dog be brought in sight of the patient he will violently attack the canine, and will then have a convulsion. It is a marked feature in cattle that there is no rise of temperature during the attack. Later, the symptoms are intensified, paralysis gradually sets in, and death relieves the patient in from four to six days.

In the horse we first notice the irritability. He will eat and drink in the early stage, but this soon becomes impossible and causes paroxysms; the victim will paw, kick, bite, strike, neigh, turn up his upper lip; nose, lips, and forelegs are the seat of intense pruritus; has increased sexual desires; will gnaw the wound, lacerate his flesh, and, during his violence, may fracture his bones, and may attack man or animals with feet and teeth. Temperature, pulse, and respirations are increased, and the attack, as in the previous cases, terminates in paralysis and death. Prior to death, spells of sweating may be noticed. · Course, four to five days.

Sheep and goats are affected in the same manner as cattle; they will stamp their feet, bleat often, have a tendency to leave their flock; will butt and hook with the horns and die in paralysis or convulsions. Course, three to four days.

The rabid pig will grunt, squeal, and perform uncontrollable movements; will hide under his litter; may attack men or animals, and has a depraved appetite; increased salivation, which is very foamy; gnaws and bites the wound. Course very rapid, death occurring in eighteen to forty-eight hours.

Poultry are attacked in a similar manner: have a period of excitement, in which they run about aimlessly; will attack man, animals, or birds with feet and bills; will attempt to eat rags, paper, etc., and, as usual, paralysis develops, and death ensues in two or three days.

*Pathology.* The lesions are extremely varied, and but a very few of them can be considered as pathognomonic. In a recent investigation[10] it was found that the most characteristic lesions were in the hippocampal region of the cerebrum, the oblongata, and the cervical myel, the cerebellum showing less marked changes. The lesions of street-rabies in the dog are more pronounced than in cases produced experimentally in rabbits, owing to the increased virulence obtained by passing the virus through several rabbits, causing a shorter period of incubation and less time for tissue changes. The changes noted were confined to the structure of the nerve-cells. It is of great importance to guard against post-mortem changes, as these may be misleading in comparison with fresh materials. It is therefore necessary to examine the tissues immediately after death, or within a few hours.

The nerve-fibres, either in the white or gray matter, undergo a certain amount of changes. The most marked alterations in the axis cylinders were revealed in the ventro-lateral columns; in some cases they were uniformly swollen throughout their entire length, while in others there were varicose enlargements. The motor-cells of the oblongata and myel in most instances show a marked vacuolation of the cell-body, as do also those of the different layers of the cerebral cortex, whereby the cytoplasm of such cells is completely usurped by the vacuolated degeneration, and in some places the partial solution of the margin of the cell gives an irregular appearance to its outline. The neuroglia cells are also found in this condition. The stimulus which is active in the destruction of the nerve fibres and cells is favorable to the production of the neuroglia, possibly contributing to the destruction of the nerve elements by their pressure.

Numerous alterations have been found in the vascular system, the most common being of an inflammatory character; proliferation of the epithelial cells and of the connective-tissue elements of the outer coat, with infiltration of lymphoid cells. These lesions may be localized at first, but later become diffuse, the changes being so great that at times the channels may be obliterated by blood-clots or hyaline masses. There are also general hyperæmia, fluidity of the blood, and lymph-stasis. Luttkemuller[6] found a moderate increase in leucocytes, accompanied by an extraordinary number of microcytes. On microscopical examination it is found that there is a migration of leucocytes in the perivascular lymphatic spaces and into the neuroglia of the nerve centres. Microscopic hemorrhages are found near the floor of the fourth ventricle, but may occur at almost any point (Horsley[6]). Hemorrhages have also been observed in the mucous membrane of the stomach, cerebrum, medulla, pons, and spinal cord. The cerebral meninges are hyperæmic, the sinuses gorged with dark blood, with minute extravasations, and there is thickening of the membranes due to interstitial fibrinous exudate and cell-production. There may be a degeneration of the cerebro-spinal epithelium and a replacement of it by blood, granular or hyaline masses. There is often congestion of the fauces, larynx, trachea, pharynx, œsophagus, and stomach.

Acute meningitis may exist with distinct effusions of lymph and even minute hemorrhages and fluid of the ventricles. Migration of white blood-corpuscles in the medulla, and an accumulation of leucocytes in the salivary glands, especially in the parotid glands, in the mucous glands of the larynx, and in the kidneys, are recorded.

Gowers[6] found pigment granules in some nerve-cells. He also writes that the principal lesions are found near the respiratory centre, hence paroxysmal spasms of the respiratory muscles.

In some instances[8] the vocal cords in cattle have been ulcerated; foreign bodies were discovered in the mouth and pharynx of dogs; the pharyngeal lymphatic glands were dark from blood engorgement.

The salivary glands are grayish-red and contain many lymphoid cells in their follicles. The stomach is congested and contracted; in the dog it contains no food, but a mixture of indigestible foreign bodies, which is considered a very characteristic symptom. There may be petechiæ or even ulcers on the mucosa. The liver is engorged, and its cells are the seat of granular degeneration. The bladder is contracted and empty, or may contain a small amount of yellow, turbid, albuminous urine.

Any of these lesions may be absent in individual cases, and others may be present. In the paralytic or lethargic forms we would not find foreign bodies in the stomach.

(To be continued.)

